# Defining the caudal limits of the endoscopic endonasal approach to the craniovertebral junction: anatomic study correlating radiographic measures

**DOI:** 10.1007/s00701-024-06389-0

**Published:** 2025-01-07

**Authors:** Mohammad Bilal Alsavaf, Moataz D. Abouammo, Jaskaran Singh Gosal, Govind S. Bhuskute, Chandrima Biswas, Guilherme Mansur, Kyle K. VanKoevering, Kyle C. Wu, Ricardo L. Carrau, Daniel M. Prevedello

**Affiliations:** 1 Department of Otolaryngology-Head and Neck Surgery, Wexner Medical Center, The Ohio State University, Columbus, OH USA; 2 Department of Neurological Surgery, Wexner Medical Center at The Ohio State University, Columbus, OH USA; 3 Department of Otorhinolaryngology-Head and Neck Surgery, Tanta University, Tanta, Egypt; 4 Department of Neurosurgery, All India Institute of Medical Sciences (AIIMS), 342005 Jodhpur, Rajasthan India; 5 Department of ENT, All India Institute of Medical Sciences, Patna, Bihar India

**Keywords:** Craniovertebral junction, Caudal limits, Radiographic anthropometric lines, Endoscopic endonasal surgery, Hard palate length, Multiport surgery

## Abstract

**Objective:**

The endoscopic endonasal approach (EEA), has become the preferred alternative to traditional open and transoral approaches to the ventral craniovertebral junction (CVJ) region. However, preoperative prediction of the limitations of caudal reach remains challenging. This cadaveric study aimed to quantify the CVJ area of exposure and access afforded by the EEA, evaluate the accuracy of previously described radiographic anthropometric lines, and identify the lowest limit of the EEA corridor.

**Methods:**

Endoscopic endonasal dissections of the CVJ were completed in 35 cadaveric specimens. The area of exposure (AoE) and caudal-most reach were measured using a navigation system. Radiographic measurements included the distance of the odontoid process from the hard palate, length of the hard palate, distance of the lowest point reached from the hard palate level, and angles such as the nasopalatine line (NPL) angle, nasoaxial line (NAxL) angle, nostril-hard palate line (NTL) angle, and rhinopalatine line (RPL) angle.

**Results:**

The mean CVJ AoE was 931.22 ± 79.36 mm2. The NPL, NAxL, and RPL angles showed significant negative correlations with the distance of the odontoid process from the hard palate line (*r* = -0.521, *p* = 0.001; *r* = -0.538, *p* = 0.001; *r* = -0.500, *p* = 0.002, respectively), while the NTL angle did not (*r* = -0.241, *p* = 0.162). No significant correlation was found between achieved AoE via EEA and NPL, NAxL, NTL, or RPL (*p* > 0.05). Importantly, hard palate length was the sole predictor of CVJ AoE variability (*r* = -0.416, *p* = 0.013), with shorter lengths associated with increased exposure. The mean distance of the lowest point reached in the AoE from the hard palate level was 9.47 ± 1.24 mm.

**Conclusions:**

This anatomic study highlights the variability in CVJ anatomy and the limitations of using previously defined radiographic anthropometric lines for predicting the caudal limits of the EEA. Hard palate length emerged as the only reliable predictor of the surgical area of exposure via the endonasal corridor. Clinical studies are warranted to validate these findings and define the potential need for adjunctive surgical routes in managing complex CVJ pathologies.

## Introduction

The craniovertebral junction (CVJ) represents an anatomically intricate region housing critical neural and vascular structures within a narrow space. Myriad pathological conditions occur in this region, including congenital abnormalities, inflammatory conditions, neoplasms, trauma, and infections [2, 16, 22]. When a ventrally located pathology results in brainstem compression or cervical myelopathy, surgical decompression via the anterior approach often becomes the definitive management strategy [6, 10].

Historically, the transoral approach has served as the primary surgical corridor for accessing the ventral CVJ and facilitating odontoid resection [3]. However, this technique comes with considerable approach-related morbidity such as dysphagia, velopharyngeal insufficiency, need for prolonged nutritional support, increased surgical site infections, and cerebrospinal fluid leak with meningitis related to traversing the contaminated oral cavity [4, 12, 17–20, 25].

In recent years, the endoscopic endonasal approach (EEA) has emerged as a minimally invasive alternative approach. By traversing the nasal cavity and nasopharynx, the EEA provides direct midline access to the CVJ while avoiding disruption of oropharyngeal mucosa. Its advantages include panoramic visualization, multi-angled instrumentation, reduced surgical morbidity, and faster recovery [8, 11, 13, 22]. Another significant advantage of EEA is its potential to preserve CVJ stability through complete or partial preservation of the C1 anterior arch, as recent evidence suggests that even partial resection can maintain biomechanical stability [21]. However, the EEA has inherent anatomical limitations, particularly in its caudal extent, constrained superiorly by the nasal bones and cartilage and inferiorly by the hard and soft palates [8, 11, 13, 22].

Due to the wide variation in the normal and pathologic anatomy of this region, accurate preoperative planning is critical to determine EEA feasibility for individual cases. Several radiographic lines have been proposed to estimate the inferior reach of the EEA preoperatively, including the nasopalatine line (NPL), nasoaxial line (NAxL), nostril-hard palate line (NTL), and more recently, the rhinopalatine line (RPL) [2, 5, 15]. However, studies suggest these lines may over- or underestimate the true caudal limits that are surgically achievable [5, 21].

This cadaveric study aims to quantify the area of exposure to the CVJ region afforded by the EEA and delineate the most accurate of the previously described radiographic lines—the NPL, NAxL, nostril, hard palate line, or RPL—to predict the true inferior extent of the EEA. By elucidating reliable radiographic predictors, we endeavor to optimize patient selection and enhance surgical planning for implementing the EEA as the sole approach to the CVJ.

## Methods

### Study design

A quantitative comparative analysis based on cadaveric dissections and anatomical measurements was conducted on 35 cadaveric specimens.

### Materials

Endoscopic endonasal dissections and radiological measurements were carried out on 35 cadaveric specimens. The cadaveric heads were fixed on the Mayfield in a natural position. For dissection, the specimens were preinjected with red latex in arteries and blue latex in veins and marked with five skull mounted screws to be used as fiducials for navigation. High-resolution computed tomography scanning was performed, and radiological data was exported to the surgical navigation system (*Stryker iNtellect image guidance).* A Mayfield head holder (*Integra Neurosciences*, New Jersey), standard endoscopic surgical instruments, high-speed endoscopic drills (Endo-Scrub2, Medtronic Surgical Technologies, Jacksonville, Florida), rod lens endoscopes with 0°, 30°, and 45° lenses (Hopkins II, 4 mm by 18 cm; Karl Storz GmbH & Co, Tuttlingen, Germany) coupled with a light source via a fiber-optic cable, a high-definition camera, and a video monitor unit (Karl Storz GmbH & Co, Tuttlingen, Germany) were utilized during the procedures. All dissections were carried out at The Ohio State University Wexner Medical Center's Anatomy Laboratory Toward Visuospatial Surgical Innovations in Otolaryngology and Neurosurgery following regulations governing the use of cadavers for research purposes. The study was exempted from the institutional review board of The Ohio State University as dissections were performed on deidentified cadavers.

DICOM reading software RADIANT (version 2024.1) was used to calculate the anatomical measurements.

### Surgical technique

#### EEA to CVJ

**Fig. 1:**
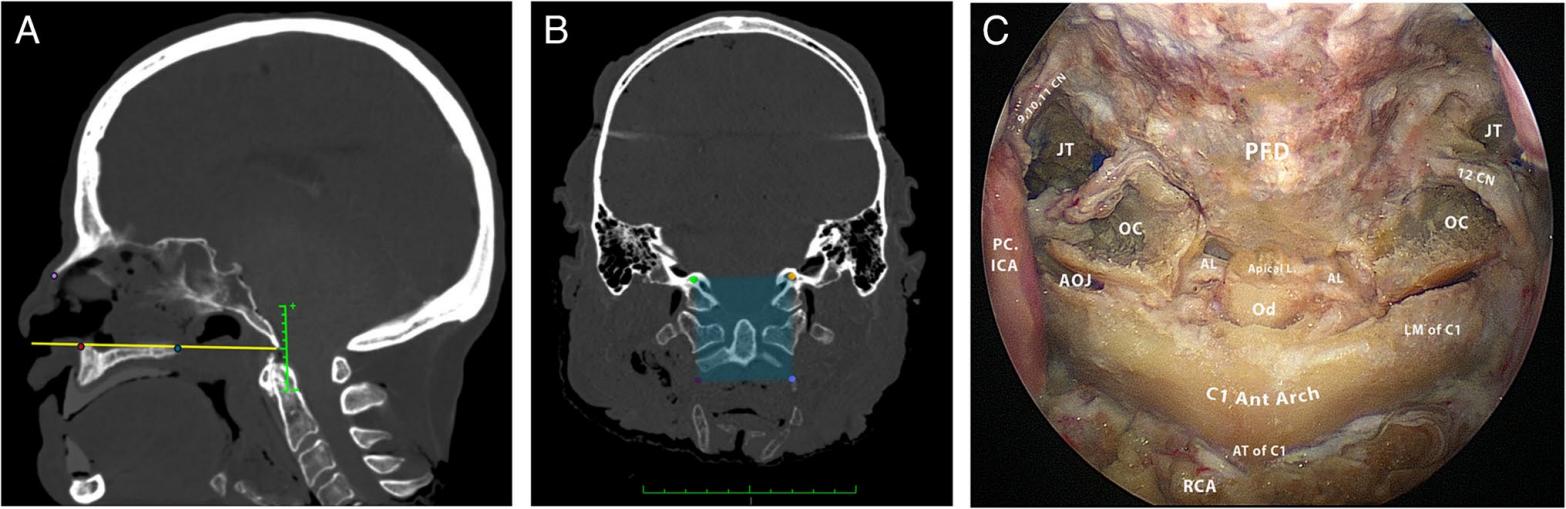
**A**. Sagittal midline view. The rhinion (pink point) marks the nasal bone tip. The hard palate is represented by a yellow line connecting the anterior (red point) and posterior (blue point) nasal spines. A green scale indicates positions relative to the hard palate, with negative values below and positive values above. **B.** Area of exposure measurement. Upper fixed points are centered on the jugular tubercles bilaterally. Lower variable points represent the maximum caudal reach of the navigation probe in the same sagittal plane as the jugular tubercles on each side. **C.** Endoscopic endonasal view of the craniovertebral junction post-dissection. Key structures labeled; PC.ICA: paraclival internal carotid artery, JT: jugular tubercle, OC: occipital condyle, AOJ: atlantooccipital joint, PFD: posterior fossa dura, Od: odontoid, Alar Lig.: alar ligament, Apical L.: Apical ligament, 9 CN: glossopharyngeal, 10 CN: vagus nerves, 11 CN: spinal accessory nerve, 12 CN: hypoglossal nerve, RCA: rectus capitis anterior, LM of C1: lateral mass of C1, AT of C1: anterior tubercle of C1

A standard endoscopic endonasal approach was performed. Initially, the inferior turbinates were lateralized, expanding the endonasal corridor. Bilateral ethmoidectomy, posterior septectomy, and sphenoidotomy with removal of the sphenoid rostrum were performed. The posterior aspect of the maxillary crest was drilled flush with the nasal floor to facilitate lateral instrument movements. The sphenoid sinus floor was drilled, and the foramen lacerum was exposed bilaterally following the vidian nerve posteriorly as described in earlier studies [14]. The clivus was drilled, and paraclival internal carotid arteries were skeletonized, exposing the superomedial petrous apex and posterior fossa dura. The pharyngobasilar fascia was incised, and the nasopharyngeal mucosa was removed to expose the prevertebral muscles along the lower clivus. The longus capitis and rectus capitis anterior muscles were reflected laterally. The jugular tubercle, hypoglossal canal, and occipital condyle, along with the anterior arch of the C1 vertebra, were exposed subperiosteally. The anterior arch of C1 was drilled to expose the odontoid. Apical and alar ligaments were identified and preserved (Fig. 1C).

### Anatomical landmarks definition

1. The **posterior nasal spine** is a bony projection at the back of the hard palate, where the left and right palatine bones meet at the midline.
2. The **anterior nasal spine** is a similar projection at the front of the maxilla, where the two maxillary bones converge at the midline. It is located at the nostrils, at the uppermost part of the philtrum.

### Quantitative Analysis

#### Distance of odontoid process from posterior nasal spine

On mid-sagittal CT imaging, the distance was measured from the “posterior nasal spine” to the odontoid tip. Measurements inferior to the hard palate line were assigned negative values, while those superior were recorded as positive values (Fig. 1A).

#### Nasopalatine line (NPL) Angle

**Fig. 2:**
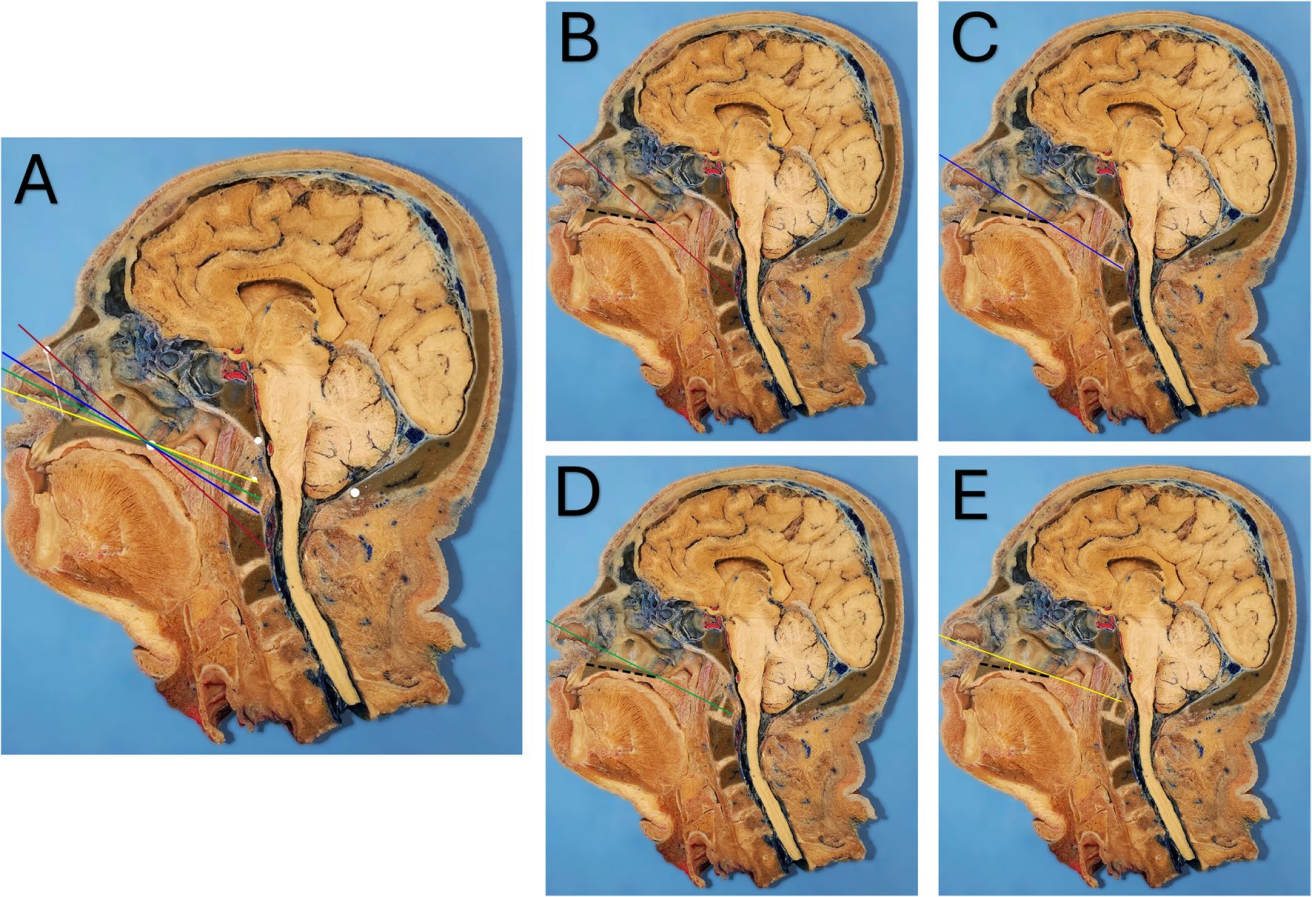
Radiographic caudal predictive lines and associated angles. **A.** Illustration of all radiographic caudal predictive lines described in the literature, showing their start points and relationships. Lines: NasoPalatine (red), NasoAxial (blue), RhinoPalatine (green), and Nostril (yellow). **B, C, D, E.** Calculated angles at the posterior nasal spine level relative to the hard palate line (dashed black line)

On mid-sagittal imaging, the NPL originates at the rhinion and extends to the posterior nasal spine tip, creating the NPL Angle (Fig. 2).

#### Nasoaxial line (NAxL) Angle

On mid-sagittal imaging, the NAxL is drawn from a midpoint between the rhinion and anterior nasal spine to the posterior nasal spine tip, creating the NAxL Angle (Fig. 2).

#### Nostril line (NTL) Angle

The NTL originates at the superior nostril margin and extends to the posterior nasal spine tip, creating the NTL Angle (Fig. 2).

#### Rhinopalatine Line (RPL) Angle

On mid-sagittal imaging, the RPL begins at two-thirds the distance from the rhinion to the anterior nasal spine and extends to the posterior nasal spine tip, creating the RPL Angle (Fig. 2).

#### CVJ area of exposure

The area of the craniovertebral junction exposed endonasally was measured in all cadavers using four anatomical landmarks. Using the navigation system, two fixed points were taken at the center of the jugular tubercle on both sides and two variable points were defined as the maximum caudal reach of the navigation probe on both sides following the same sagittal plane of the jugular tubercles. Those two variable points made the difference in the area measured on each cadaver. The X, Y, and Z coordinates for each of the four points were recorded and employed in Heron’s formula to calculate the total area of exposure in mm^2^ [24] (Fig. 1B).

### Statistical analysis

This study presents the means and standard deviations for continuous variables that followed an approximately normal distribution. Simple linear regression was used to assess the strength and direction of the relationship (Pearson's r) between continuous parameters. A significance level of 0.05 was applied as the threshold for determining statistical significance across all tests. Statistical analyses were performed using JASP version 0.18.3.

## Results

### Mean craniocervical measurements

The mean distance of the odontoid process tip from the hard palate line level was −5.67 ± 6.42 mm. The mean distance from the posterior nasal spinal tip to the anterior tubercle of C1 was 32.53 ± 3.84 mm, and the mean hard palate length was 49.0 ± 4.78 mm. The mean angles measured were: NPL angle 29.0° ± 4.52°, NAxL angle 17.47° ± 3.48°, NTL angle 14.43° ± 3.81°, RPL angle 12.55° ± 2.65°. The mean craniovertebral junction (CVJ) area of exposure was 931.22 ± 79.36 mm^2^ (Table 1).

**Table 1:** Quantitative measurements obtained from CT scan images and navigation system

Measurements	Mean (SD)
Distance of Odontoid Process from Hard Palate Level (mm)	−5.67 (6.42)
Distance from Posterior Nasal Spine to C1 Arch (mm)	32.53 (3.84)
Hard Palate Length (mm)	49 (4.78)
NPL Angle (degree)	29 (4.52)
NAxL Angle (degree)	17.47 (3.48)
NTL Angle (degree)	14.43 (3.81)
RPL Angle (degree)	12.55 (2.65)
CVJ Area of Exposure (mm^2^)	931.22 (79.36)
Distance of Lowest Point Reached from the Hard Palate Level (mm)	9.47 (1.24)

### Relationship between odontoid tip position and predictive lines

The NPL angle (*r* = −0.521, *p* = 0.001), NAxL angle (*r* = −0.538, *p* = 0.001), and RPL angle (*r* = −0.500, *p* = 0.002) showed significant negative correlations with the distance of the odontoid process from the hard palate line, meaning that as the distance between the nasal bone and the anterior nasal spine tip increases, the odontoid is located at a lower level. Conversely, as this distance decreases, the odontoid is positioned closer to the hard palate line level or above. However, the NTL angle did not demonstrate a significant correlation (*r* = −0.241, *p* = 0.162) with the distance of the odontoid process from the hard palate line (Table 2).

**Table 2:** Correlation analysis between odontoid tip position and established radiographic caudal predictive lines

	Pearson’s r	95% CI		p-value
		Lower	Upper	
**NPL Angle**	−0.521	−0.728	−0.221	**0.001**
**NAxL Angle**	−0.538	−0.739	−0.249	**0.001**
**NTL Angle**	−0.241	−0.532	0.100	0.162
**RPL Angle**	−0.500	−0.714	−0.201	**0.002**

### Measurements influencing craniovertebral junction area of exposure

A significant negative correlation was found between the hard palate length and CVJ's area of exposure (*r* = −0.416, *p* = 0.013), indicating that for every 1 mm decrease in hard palate length, the CVJ's area of exposure increased by an average of 6.914 mm^2^. No other significant correlations were observed between the CVJ's area of exposure and distance from the hard palate to the C1 arch, NPL angle, NAxL angle, NTL angle, RPL angle (Table 3).

**Table 3:** Simple linear regression analysis of factors influencing the craniovertebral junction area of exposure

	Pearson’s r	95% CI		p-value
		Lower	Upper	
**Distance from Posterior Nasal Spine to C1 Arch**	−0.110	−0.427	0.232	0.531
**Hard Palate Length**	−0.416	−0.658	−0.079	**0.013**
**NPL Angle**	0.155	−0.187	0.465	0.372
**NAxL Angle**	0.068	−0.272	0.392	0.699
**NTL Angle**	−0.019	−0.350	0.316	0.914
**RPL Angle**	0.042	−0.295	0.370	0.810
**Naso-Odontoid-Palatine Angle**	−0.129	−0.444	0.213	0.459
**Naso-Basion-Palatine Angle**	−0.113	−0.430	0.228	0.517
**Naso-Opisthion-Palatine Angle**	−0.123	−0.439	0.219	0.480

The mean distance of the lowest point reached in the AoE from the hard palate level was 9.47 ± 1.24 mm. A significant negative correlation was observed between the hard palate length and the ultimate lowest reach through an endonasal approach, (*r* = −0.339, *p* = 0.047). This indicates that for every 10 mm decrease in hard palate length, the furthest lower reach increased by an average of 7.7 mm.

## Discussion

This anatomic study shows that the hard palate length (from the anterior nasal spine to the posterior nasal spine) is the single most important criterion determining both the exposure area of CVJ and the caudal reach of the EEA. The shorter the hard palate length, the greater the area exposed around CVJ, and the greater the inferior exposure via EEA.

The intricate anatomy, deep central location, and vital neurovascular structures traversing the CVJ render it one of the most surgically formidable regions to access. An additional challenge is the substantial congenital variation and anatomical diversity observed in this area, such as platybasia and variable odontoid process length and positioning relative to the hard palate [16]. Furthermore, the inherent mobility of the CVJ and pathologies like congenital atlantoaxial dislocation and basilar invagination, which usually consist of atlantooccipital assimilation/variable odontoid length well above the level of the basion, underscores the need to consider multiple individualized factors when formulating treatment strategies. Historically, open transcervical and transoral approaches served as the primary surgical corridors, [3] subsequently supplanted by the EEA which confers significantly reduced morbidity and mortality rates [8, 11, 13, 22]. Nevertheless, the EEA has intrinsic limitations in its caudal exposure, constrained by nasal bones and cartilages anteriorly and by the hard and soft palate posteriorly.

Several radiographic lines have been proposed in the past to predict the inferior extent of the EEA corridor. Accurate preoperative estimation is crucial to determine the surgical boundaries and the potential need for adjunctive surgical routes, such as posterior palatectomy or the recently described multiport corridors via contralateral nasofrontal trephination or contralateral medial transorbital [1, 7, 9]. Initially, the NPL was defined by Almeida et al [5]; however, Aldana et al. subsequently noted overestimation by this line and introduced two novel measures: the NTL and NAxL [2]. They found the NTL to underestimate the surgical limit while emphasizing the precise predictive value of the NAxL. After three years, they re-evaluated additional cases and proposed the RPL as the most accurate predictor, suggesting the NAxL overestimated the inferior boundary [15]. Interestingly, in our study of 35 cadaveric specimens, we found the mean RPL angle to be smaller than the previously discouraged NTL due to its underestimation (Table 1).

The underlying reason for this discrepancy likely stems from inter-individual anatomical variations, especially between the odontoid tip to the level of the hard palate. We observed that as the odontoid process migrated superiorly relative to the hard palate plane, the angles created by the NPL, NAxL, and RPL significantly decreased. More interestingly, we observed a reduction in the distance from the anterior nasal spine to the rhinion as the odontoid elevated above the level of the hard palate, although the reason and significance of this phenomenon remain unclear. Thus, relying solely on the previously described radiographic lines will not give an accurate preoperative estimation of the exposed CVJ area. Hence, instead of relying on odontoid positioning, we quantified the area of exposure (AoE) to the CVJ achieved via the endoscopic endonasal dissection in each cadaveric specimen. Our analysis revealed that hard palate length was the sole predictor of CVJ AoE variability. (Table 3).

As per our results in 35 cadaveric specimens, the mean distance of the lowest point reached in the AoE through the endonasal approach from the hard palate level was 9.47 ± 1.24 mm. This measurement might be marginally higher in real cases because of increased tissue rigidity of the soft palate encountered in fixed cadaveric specimens. Importantly, this study identified hard palate length as the only reliable predictor of CVJ AoE. These findings corroborate previous data from our institution, which demonstrated that posterior palatectomy substantially enhanced caudal access when employing both straight and angled drills [7, 23]. Our surgical technique begins with a straight drill, preserving the integrity of the soft palate and oral mucosa. This subsequently allows us to drop angled drills and extend our inferior reach.

Further prospective clinical studies are warranted to substantiate these findings. Moreover, additional comparative cadaveric investigations evaluating potential adjunctive surgical corridors are needed to define the maximum achievable AoE or caudal surgical limit when implementing multiport approaches.

## Limitations

This cadaveric study underscores the variation in CVJ anatomy and the limitations of using previously defined lines for the odontoid. However, the endonasal approach's caudal reach might be less in cadavers compared to live tissue due to the fixative process.

Our simple regression model suggests that hard palate length is a significant predictor of the distance extending below the palate. However, low R^2^ value (0.115) indicates that other unmeasured factors likely play substantial roles in determining this anatomical relationship. This highlights the complex nature of craniofacial structures and the need for more comprehensive models in future research.

## Conclusions

This study elucidated challenges in predicting the caudal exposure of the EEA to the CVJ due to substantial anatomic variability in this region. The inverse relationship between the height of the odontoid process and the distance between the nasal bone and anterior nasal spine exemplifies one aspect of these challenges. Previously described radiographic lines demonstrated inconsistent correlations with the inferior limits achievable endonasally. The hard palate length emerged as the sole reliable predictor of the CVJ area of exposure attainable via EEA, with shorter lengths affording greater caudal reach. Further clinical studies are needed to validate implementing adjunctive surgical corridors to extend the inferior boundaries beyond the endonasal constraints.

## Data Availability

No datasets were generated or analysed during the current study.

## Abbreviations

*AoE*: Area of Exposure
*CVJ*: Craniovertebral Junction
*EEA*: Endoscopic Endonasal Approach
*NPL*: Nasopalatine Line
*NAxL*: Nasoaxial Line
*NTL*: Nostril-Hard Palate Line
*RPL*: Rhinopalatine Line
*CT*: Computed Tomography
